# A Phylogenomic Analysis of the Floral Transcriptomes of Sexually Deceptive and Rewarding European Orchids, *Ophrys* and *Gymnadenia*

**DOI:** 10.3389/fpls.2019.01553

**Published:** 2019-11-29

**Authors:** Laura Piñeiro Fernández, Kelsey J. R .P. Byers, Jing Cai, Khalid E. M. Sedeek, Roman T. Kellenberger, Alessia Russo, Weihong Qi, Catharine Aquino Fournier, Philipp M. Schlüter

**Affiliations:** ^1^ Institute of Botany, University of Hohenheim, Stuttgart, Germany; ^2^ Department of Systematic and Evolutionary Botany, University of Zurich, Zurich, Switzerland; ^3^ Department of Zoology, University of Cambridge, Cambridge, United Kingdom; ^4^ Center for Ecological and Environmental Sciences, Northwestern Polytechnical University, Xi’an, China; ^5^ Laboratory for Genome Engineering and Synthetic Biology, Division of Biological Sciences, King Abdullah University of Science and Technology, Thuwal, Saudi Arabia; ^6^ Agricultural Genetic Engineering Research Institute (AGERI), Agriculture Research Centre, Giza, Egypt; ^7^ Department of Plant Sciences, University of Cambridge, Cambridge, United Kingdom; ^8^ Department of Plant and Microbial Biology, University of Zurich, Zurich, Switzerland; ^9^ Functional Genomics Centre Zurich, Zurich, Switzerland

**Keywords:** phylogenomics, orchids, *Ophrys*, *Gymnadenia*, transcriptome, pollination strategy

## Abstract

The orchids (Orchidaceae) constitute one of the largest and most diverse families of flowering plants. They have evolved a great variety of adaptations to achieve pollination by a diverse group of pollinators. Many orchids reward their pollinators, typically with nectar, but the family is also well-known for employing deceptive pollination strategies in which there is no reward for the pollinator, in the most extreme case by mimicking sexual signals of pollinators. In the European flora, two examples of these different pollination strategies are the sexually deceptive genus *Ophrys* and the rewarding genus *Gymnadenia*, which differ in their level of pollinator specialization; *Ophrys* is typically pollinated by pseudo-copulation of males of a single insect species, whilst *Gymnadenia* attracts a broad range of floral visitors. Here, we present and describe the annotated floral transcriptome of *Ophrys iricolor*, an *Andrena-*pollinated representative of the genus *Ophrys* that is widespread throughout the Aegean. Furthermore, we present additional floral transcriptomes of both sexually deceptive and rewarding orchids, specifically the deceptive *Ophrys insectifera*, *Ophrys aymoninii*, and an updated floral transcriptome of *Ophrys sphegodes,* as well as the floral transcriptomes of the rewarding orchids *Gymnadenia conopsea, Gymnadenia densiflora, Gymnadenia odoratissima*, and *Gymnadenia rhellicani* (syn. *Nigritella rhellicani*). Comparisons of these novel floral transcriptomes reveal few annotation differences between deceptive and rewarding orchids. Since together, these transcriptomes provide a representative sample of the genus-wide taxonomic diversity within *Ophrys* and *Gymnadenia* (Orchidoideae: Orchidinae), we employ a phylogenomic approach to address open questions of phylogenetic relationships within the genera. Specifically, this includes the controversial placement of *O. insectifera* within the *Ophrys* phylogeny and the placement of “*Nigritella*”-type morphologies within the phylogeny of *Gymnadenia*. Whereas in *Gymnadenia*, several conflicting topologies are supported by a similar number of gene trees, a majority of *Ophrys* gene topologies clearly supports a placement of *O. insectifera* as sister to a clade containing *O. sphegodes*.

## Introduction

Orchidaceae and Asteraceae constitute the largest families of flowering plants. Over 800 orchid genera and 25,000 species have been described, with an average rate of 500 species and 13 genera described per year (Cribb et al., 2003; Chase et al., 2015). Orchids have colonized a great variety of geographical ranges, from Scandinavia to Tierra del Fuego (Antonelli et al., 2009; Domínguez and Bahamonde, 2013), although the vast majority of species occur in tropical and neotropical areas (Dressler, 1993). The key to their success has variously been hypothesized to reside in their epiphytic habitat (for tropical orchids) or in their high level of pollinator specialization (Gravendeel et al., 2004; Cozzolino and Widmer, 2005). About two thirds of orchid species present rewards to their visitors, in most cases, nectar (Dafni and Ivri, 1979; Bell et al., 2009; Johnson et al., 2013). These rewarding species are commonly generalized in their pollination, attracting a wide range of pollinators (Brantjes, 1981; Claessens and Kleynen, 2017). However, the ability to produce nectar is missing in one third of species across the family. Instead, they have developed alternative mechanisms based on deception (Ackerman, 1986; Jersáková et al., 2006; Schiestl and Schlüter, 2009; Johnson and Schiestl, 2016). Some of these mechanisms target generalist pollinators, e.g., food deception, where orchids attract pollinators by advertising floral cues that resemble those from rewarding plants (Salzmann et al., 2007; Braunschmid et al., 2017). On the other hand, orchids have also developed mechanisms such as sexual deception to attract highly specialized pollinators. Sexually deceptive flowers produce chemical signals that mimic the sexual pheromones of pollinators, and thus, lead the pollinators to “pseudo-copulate“ with the flowers (Kullenberg and Bergström, 1976; Paulus and Gack, 1990; Schiestl et al., 1999). Examples of such behaviour occur in the Australian *Chiloglottis* spp. (Mant et al., 2002; Schiestl et al., 2003), or the recently discovered sexually deceptive *Caladenia abbreviata* (Phillips and Peakall, 2018).

In the European flora, one can find representatives of the aforementioned pollination strategies in the sexually deceptive genus *Ophrys* and the rewarding genus *Gymnadenia,* both within the subtribe Orchidinae (subfamily Orchidoideae) (Inda et al., 2012). Orchids from the Mediterranean genus *Ophrys* attract male pollinators by means of sexual deception (Paulus and Gack, 1990; Ayasse et al., 2000; Schiestl et al., 2000). Attractiveness to pollinators in the genus is highly species-specific, that is, each *Ophrys* species normally attracts a single pollinator species (Paulus and Gack, 1990; Paulus, 2018) by releasing chemicals (for solitary bees, mostly alkenes) mimicking the female sex pheromones (Schiestl et al., 2000; Schlüter and Schiestl, 2008; Xu et al., 2012). This high specificity acts as a pre-zygotic barrier and facilitates reproductive isolation between orchid species (Xu et al., 2011; Xu et al., 2012; Paulus, 2018). *Ophrys* is a recently diverged genus (crown age estimated ca. 5 Ma) with ancestral wasp pollination (Breitkopf et al., 2015), but extant species are commonly pollinated by solitary bees, e.g. *Eucera* or *Andrena* (Paulus and Gack, 1990; Gaskett, 2011). Successful floral isolation and species divergence in the genus may easily be achieved by shifts between similar pollinators, where small changes in genes involved in the pheromone profiles can lead to attraction of new, related pollinators (Schlüter et al., 2011; Sedeek et al., 2014; Schlüter, 2018). For instance, after two independent shifts to (mostly) *Andrena* solitary bee pollination (Breitkopf et al., 2015), two parallel adaptive radiations have taken place simultaneously within the last ca. 1 Ma, yielding two major clades, the *Ophrys sphegodes* and the *Ophrys fusca* species complexes. In line with its recent radiation, a large amount of genetic polymorphism is shared across closely related species within the *O. sphegodes* complex, which has been attributed to common ancestry rather than independent mutations or recent hybridization, although a hybridization event *prior* to radiation seems distinctly possible (Sedeek et al., 2014; Roma et al., 2018; Cozzolino et al., 2019). Coalescence theory predicts that in the case of a radiation, the time of coalescence of these polymorphic alleles will often predate the split of species (Takahata, 1989). Yet, or maybe because of this, phylogenetic relationships within *Ophrys* remain controversial, with different markers in the genome potentially painting different pictures of relationships (Cozzolino et al., 2019). Phylogenetically, the ca. 10 main *Ophrys* lineages are split into three major clades (where clade α includes *Ophrys insectifera*, β includes the *O. fusca s.l.* lineage and γ includes the *O. sphegodes s.l.* lineage) and the relationships among major lineages within these clades are relatively clear, although one major question remains unclear. In particular, the placement of the wasp-pollinated *O. insectifera* L. (clade α) within the *Ophrys* phylogeny has been suggested to be either the earliest-branching lineage [topology: (*α,*(*β,γ*))] or more closely related to the *O. sphegodes* lineage [topology: (*β,*(*α,γ*))] (cf. e.g. Breitkopf et al., 2015; Bateman et al., 2018b, and references therein).

The Eurasian genus *Gymnadenia* is characterized by fragrant, purple to white, resupinate flowers that mainly attract diurnal and nocturnal Lepidoptera species offering nectar as a reward. Although they attract a wide range of Lepidoptera, and some species are found in sympatry, pollinator overlap is minimal between most species (Vöth, 2000; Huber et al., 2005; Claessens and Kleynen, 2011) and strong pollinator-mediated reproductive isolation has been reported between the putative sister species *G. odoratissima* (L.) Richard and *Gymnadenia conopsea* (L.) Brown (Sun et al., 2015). The latter species is strongly genetically differentiated from the morphologically similar taxon *G. densiflora* (Wahlenberg) Dietrich (Stark et al., 2011). Finally, the Alpine *G. rhellicani* (Teppner & E. Klein) Teppner & E. Klein (syn. *Nigritella rhelliani*) represents a morphologically distinct lineage within the genus, characterized by extremely dense inflorescences, generally dark red and without resupination, i.e. the labellum remains pointing upwards as opposed to rotated downwards as in other *Gymnadenia* species. The former genus *Nigritella* was merged into *Gymnadenia* only following molecular phylogenies (Hedrén et al., 2000). Previous phylogenetic analysis have shown that *Gymnadenia odoratissima* is sister to *Gymnadenia conopsea*, and *Gymnadenia densiflora* forms a clade with *Gymnadenia rhellicani* (Bateman et al., 2003; Sun et al., 2015). However, these relationships remain contentious, since other studies support a sister-group relationship among *Nigritella* and the “classical” genus *Gymnadenia* (Hedrén et al., 2000; Brandrud et al., 2019). Hence, further attention is warranted, especially to clarify the position of *Nigritella*. The age of the most recent common ancestor shared among all *Gymnadenia/Nigritella* species is estimated to be around 2.5–3 Ma (Inda et al., 2012).

Due to the high taxonomic complexity of Orchidaceae, reconstructing phylogenetic patterns to understand relationships in the family remains challenging. In the last decades, phylogenetic studies in orchids moved from a morphological (Chittka and Menzel, 1992; Gravendeel et al., 2004) to a molecular approach aiming to provide a better insight into orchid relationships (Cameron et al., 1999; Stark et al., 2011; Inda et al., 2012; Breitkopf et al., 2015; Givnish et al., 2015; Bateman et al., 2018a). Previously, the focus of these analyses was at the level of using few genetic markers, e.g. ITS, to reconstruct phylogenies. However, this approach can be problematic as some markers are chosen by their relevance or suitability in a certain taxonomic group, even though they could present low resolution for certain taxonomic groups (Capella-Gutiérrez et al., 2014). Moreover, this approach generally focuses on estimating one coherent tree (e.g. by concatenating sequences), which ignores the fact that different loci can have different phylogenetic histories. Especially when dealing with recently diverged groups with incomplete lineage sorting (Pamilo and Nei, 1988), a genomic approach focusing on understanding patterns on different gene genealogies, may allow the quantification of the different phylogenetic scenarios and thus, be more informative on the evolutionary history of a group (Pease and Hahn, 2015; Pease et al., 2016). Orthologous genes, described as homologous genes that originated from a common ancestral gene as a result of the speciation process (Fitch, 1970), tend to retain the original function from the common ancestor over evolutionary time (Jensen, 2001). Thus, groups of orthologous genes within gene families, together with a genome-wide approach, are perfect candidates to resolve orchid phylogeny and effectively clarify their relationships in an evolutionary framework (Li et al., 2003; Deng et al., 2015).

Here, we present the novel floral transcriptome of the Mediterranean sexually deceptive orchid *Ophrys iricolor* Desf., a representative of the genus *Ophrys* in the Aegean area, which is considered to be a member of the *O. fusca* group (clade β) and represents the evolutionarily distinct abdomen-pollinated members of the genus (previous section *Pseudophrys*) (Schlüter et al., 2009). In addition, we present several floral transcriptomes of both rewarding and deceptive orchids of the subtribe Orchidinae, particularly the rewarding orchids *G. conopsea, G. densiflora, G. odoratissima*, and *G. rhellicani*, together with the sexually deceptive *O. insectifera*, *Ophrys aymoninii* (Breistroffer) Buttler, and finally, an updated transcriptome of *O. sphegodes s.l.* (Sedeek et al., 2013). Using a set of orthologous genes, we employ a genome-wide approach to phylogenetic analysis of these novel floral transcriptomes together with published orchid transcriptomic/genomic data, to compare the transcriptomes of deceptive and rewarding orchids. Furthermore, as these transcriptomes cover the genus-wide taxonomic diversity within *Ophrys* and *Gymnadenia*, our objectives are to elucidate (1) the placement of the *O. insectifera* complex within the three major clades in the *Ophrys* phylogeny, (2) the placement of the morphologically distinct *G. rhellicani* (and presumably other members of subgenus *Nigritella*) within the phylogeny of *Gymnadenia* and (3) whether there is evidence of introgression due to shared pollinators in distinct *Ophrys* lineages.

## Material and Methods

### Plant Material

The novel *Ophrys iricolor s.l.* (*O. iricolor s.s.* and *Ophrys mesaritica* H.F. Paulus, C. Alibertis & A. Alibertis) cross-species transcriptome is presented here. Data from the putative sister species *O. iricolor s.s.* and *O. mesaritica* (Schlüter et al., 2009) were assembled into a single transcriptome due to expected high levels of allele sharing among the group, as seen in the *O. sphegodes* complex (Sedeek et al., 2013; Sedeek et al., 2014). Sample size (*Ophrys iricolor s.l.,* N = 16 biological replicates; *O. sphegodes s.l.,* N = 37) and provenance are listed in **Table 1**. The previously published cross-species *O. sphegodes s.l.* (*O. exaltata* subsp. *archipelagi* (Gölz & H.R. Reinhard) Del Prete, *O. garganica* Nelson ex O. & E. Danesch, and *O. sphegodes* Miller) transcriptome (Sedeek et al., 2013) is here updated with data from additional samples, including from *O. incubacea* Bianca (samples from Sedeek et al., 2014) within the same species complex that is characterized by the aforementioned high levels of allele and transcript sharing among species (Sedeek et al., 2013; Sedeek et al., 2014) and is hence covered in a single cross-species transcriptome assembly. Additionally, *O. insectifera* and *O. aymoninii* transcriptomes are also presented here. Data from *O. insectifera* and *O. aymoninii* (collected in Gervasi et al., 2017), were assembled into separate transcriptomes because these species are pollinated by different types of pollinators (*O. insectifera* is wasp-pollinated, while *O. aymoninii* is *Andrena*-pollinated) and the assumption of high levels of within-group allele sharing cannot be made. Finally, sampled flowers from the clearly distinct species *G. conopsea, G. densiflora, G. odoratissima* and *G. rhellicani* (from Kellenberger et al., 2019) were used to create individual transcriptome assemblies for these species to complement the published cross-species *Gymnadenia* transcriptome assembly (N = 10, **Table 1**) (Kellenberger et al., 2019). As far as it was possible to ascertain pollination status (not always possible for *Gymnadenia* flowers), all samples used in this study were from unpollinated flowers of diploid individuals. Flowers were flash-frozen and stored at ‑80°C until RNA extraction was conducted as detailed by Kellenberger et al. (2019). Since polyploids are known from *Gymnadenia* and (occasionally) *Ophrys* and to ensure that all samples sequenced were diploid, ploidy levels of *O. iricolor* and *O. mesaritica* were checked *via* flow cytometry of pollinia as described by Xu et al. (2011) using a Cell Lab Quanta™ SC-MPL flow cytometer (Beckman Coulter, Fullerton, Canada)*. Phaseolus coccineus* “Scarlett Emperor” (sativa Rheinau SG, Switzerland) leaf material was used as internal standard. Ploidy levels were previously described for *O. sphegodes s.l.* (Sedeek et al., 2014)*, O. insectifera* and *O. aymoninii* (Gervasi et al., 2017) and the four *Gymnadenia* species (Kellenberger et al., 2019) used in this study, including all sequenced individuals.

**Table 1:** Statistics of transcriptomic data for each species/assembly.

Assembly	*O. iricolor s.l.* complex	*O. sphegodes s.l.* complex v.2	*O. insectifera*	*O. aymoninii*	*Gymnadenia* spp. cross-species assembly^3^	*G. conopsea*	*G. densiflora*	*G. odoratissima*	*G. rhellicani*
									
Number of biological samples^1^	16 (I: 8, M: 8)^a,b^	37 (E: 9, G: 10, I: 8, S: 10)^c,d,e^	1	1	10 (C: 1; D: 2; O: 1; R: 6)^f^	1	2	1	6
Sample origin^2^	this study: Crete, Greece^g^	R1, R2	R3	R3	R4	R4	R4	R4	R4
Assembly^2^	this study	v1: R5, v2: this study	this study	this study	R4	this study^h^	this study^h^	this study^h^	this study^h^
Illumina Technology	HiSeq 2000 (PE100)	HiSeq 2000 (PE100)	HiSeq 2000 (PE100)	HiSeq 2000 (PE100)	HiSeq 2500 (PE125)	HiSeq 2500 (PE125)	HiSeq 2500 (PE125)	HiSeq 2500 (PE125)	HiSeq 2500 (PE125)
Number of PE reads	493 522 864	1 340 285 065	43 629 062	41 727 306	191 906 267	8 364 102	45 753 532	21 459 264	116 329 369
Sequenced bases (Gbp)	98.7	268.1	8.7	8.3	48.0	2.1	11.4	5.4	29.1
Number contigs	131 528	547 360	81 951	66 505	589 218	100 467	255 230	144 454	430 600
GC%	42.41	41.41	44.76	44.97	44.22	46.45	45.37	44.10	44.08
N50 length	1018	973	1107	1200	553	1152	1295	1126	826
SRA accessions	PRJNA574279	PRJNA574279	PRJNA574279	PRJNA574279	PRJNA504609	PRJNA504609	PRJNA504609	PRJNA504609	PRJNA504609
TSA accession number	GHXI00000000	GHXJ00000000	GHWX00000000	GHWW00000000	figshare: 7314731 ^i^	GHXG00000000	GHXE00000000	GHXF00000000	GHXH00000000

^1^Generally, for Ophrys, one biological sample refers to one fresh, anthetic unpollinated flower labellum of one plant individual collected in the field, except as detailed under note **^d^** for O. sphegodes s.l. v.2, or, for Gymnadenia, to a small number of anthetic flowers;

^2^References are R1: Schlüter et al. (2011), R2: Sedeek et al. (2014), R3: Gervasi et al. (2017), R4: Kellenberger et al. (2019), R5: Sedeek et al. (2013);

^3^Raw sequencing data for this column represents the sum of data from all Gymnadenia samples;

^a^Species are I: O. iricolor, M: O. mesaritica; ^b^one O. iricolor sample failed to produce results; ^c^species are E: O. exaltata subsp. archipelagi, G: O. garganica, I: O. incubacea, S: O. sphegodes; ^d^for O. exaltata, O. garganica and O. sphegodes, one sample each was derived from labella at bud stage (same biological individuals as used for open flowers), and one sample each of these species was field-collected whereas the remaining samples were grown under greenhouse conditions (Schlüter et al., 2011); ^e^for O. incubacea, 3 were sampled under greenhouse conditions and 5 were collected in the field and were added in a second sequencing batch; ^f^species are C: G. conopsea, D: G. densiflora, O: G. odoratissima, R: G. rhellicani; ^g^O. mesaritica was sampled at Pirgos, Crete (28 February 2013; accessions PMS540 A,D,K,N,O,Q,R,T) and O. iricolor at Kato Chorio, Crete (8 April 2013; accessions PMS558 E, I), Vasiliki, Crete (9 April 2013; PMS560 A, C) and at Jouchtas, Crete (10 April 2013; PMS561 A, I, H, O), all under permit number 125001/95 issued on 28 January 2013 by the Hellenic Republic Ministry Of The Environment, Energy & Climate Change, Athens, Greece. ^h^Raw sequencing data published by Kellenberger et al. (2019). ^i^Figshare rather than TSA identifier.

### RNA Extraction, Library Preparation and Sequencing

Total RNA was extracted separately for each biological individual and tissue with TRIzol reagent (Thermo Fisher Scientific, Massachusetts) according to the manufacturer's protocol followed by a purification step using Qiagen RNeasy MinElute Cleanup Kit (Qiagen, Netherlands). Quality of the isolated RNA was determined with a Qubit^®^ (1.0) Fluorometer (Life Technologies, California, USA) and a Bioanalyzer 2100 (Agilent, Waldbronn, Germany). Paired-end sequencing was performed on the Illumina HiSeq 2000 or 2500 platforms (Illumina, Inc, California, USA) for *Ophrys* and *Gymnadenia* samples (**Table 1**), generating separate files for each biological sample.

### Transcriptome Assemblies and Functional Annotation

**Figure 1:**
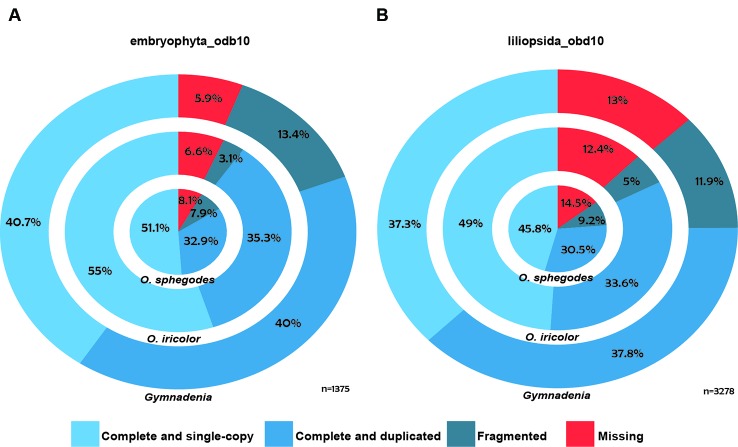
BUSCO assessment. Concentric circles show the BUSCO assessment of *O. sphegodes* v.2, *O. iricolor* and cross-species *Gymnadenia* spp. transcriptomes (from inside to outside), where the first three (blue) categories together are taken as an estimation of transcriptome “completeness”. **(A)** BUSCO results with embryophyta_odb10 and **(B)** the larger liliopsida_odb10 databases.

Individual reads were first aligned to PhiX Control library (Illumina) sequences using bowtie2 v2.2.4 (Langmead and Salzberg, 2012) to remove sequencing control reads. Filtered reads were trimmed using Trimmomatic v. 0.36 (Bolger et al., 2014) to remove any Illumina adapters. Surviving reads were then de-novo assembled to transcripts using Trinity r20140717/v. 2.0.618 (Grabherr et al., 2011). In the case of *O. sphegodes*, where a previous assembly based on 454, Solexa and Sanger data was available (Sedeek et al., 2013), additional Illumina HiSeq reads were assembled with Trinity as described above and then merged with the published assembly using cd-hit-est (Li and Godzik, 2006; Fu et al., 2012) (95% sequence identity threshold with full length alignment coverage for the shorter sequence). Protein coding regions were analysed using TransDecoder r20140704 (http://transdecoder.github.io) (Haas et al., 2013). The assembled contigs were annotated with the standard Trinotate annotation pipeline (https://trinotate.github.io/) (Grabherr et al., 2011) against Swissprot (Boeckmann et al., 2003), Pfam (Finn et al., 2014), TmHMM (Krogh et al., 2001), Gene Ontology (Ashburner et al., 2000) and SignalP (Petersen et al., 2011). Due to high levels of overlap among the four single-species *Gymnadenia* transcriptomes (**Figure S1B**), we annotated only the cross-species *Gymnadenia* transcriptome from all four species. For purposes of comparison, we also updated the annotation of the previously published, updated (v.2) transcriptome of *O. sphegodes* (Sedeek et al., 2013) with Trinotate. Finally, to estimate the completeness of the transcriptomes, we performed a BUSCO v3.1.0 assessment (Simão et al., 2015) with the lineage databases embryophyta_odb10 and liliopsida_odb10 (**Figures 1A**, **B**).

### Phylogenomic Analysis

OrthoMCL v2.0.9 (Li et al., 2003) was used under the MySQL v14.14 server to identify orthologous groups based on annotated coding sequences (CDS) (where no annotated CDS were available, they were derived by TransDecoder as above) of 15 members of the Orchidaceae family including the above described *Ophrys* and the four *Gymnadenia* single-species transcriptome assemblies together with the transcriptomes/genomes of *Apostasia shenzhenica* and *Phalaenopsis equesteris* (Zhang et al., 2017)*, Dactylorhiza fuchsii* (Balao et al., 2017)*, Chiloglottis trapeziformis* (Wong et al., 2017)*, Dendrobium catenatum* (Zhang et al., 2016), and *Platanthera clavellata* and *Goodyera pubescens* (retrieved from the 1KP project; http://www.onekp.com/). Following the TranslatorX pipeline (Abascal et al., 2010), sequences were aligned using Mafft v7.407 (Katoh and Standley, 2013). To construct phylogenetic trees, a pipeline as described in Xu et al. (2017) was followed. In brief, poorly aligned sequences were removed using trimal v1.2 (Capella-Gutiérrez et al., 2009). Selection of the best-fit models of nucleotide substitution was performed with jModelTest 2.1.10 (Santorum et al., 2014), with parameters: -f -i -g 4 -a -AIC -s 3. This allowed the inclusion of models with unequal base frequencies, a proportion invariable sites, rate variation among sites and set 4 categories, model-averaged phylogeny for each active criterion. Moreover, it used AIC (Akaike Information Criterion) for model selection and accounted for 3 substitution schemes. Maximum likelihood trees of the best-fit models were calculated with phyML 3.3 (Guindon and Gascuel, 2003). For each taxonomically fully sampled orthologous group, tree topologies from *Ophrys* and *Gymnadenia* single-copy gene branches were extracted. In addition, we also extracted topologies where one *Ophrys* species was missing. The extraction of tree topologies was automated with an in-house R script. Moreover, for both *Ophrys* and *Gymnadenia*, we extracted topologies where gene duplications happened only within a monophyletic group of a given species. In the latter case, all but one of the duplicate tips was dropped from the phylogeny (keep.tip function from the package ape for R v3.5.0) (R Core Team, 2001). After retrieving (rooted) topologies of target groups, we compared these topologies with Robinson-Foulds distances, where a distance of 0 indicates that topologies are in full agreement with each other (Robinson and Foulds, 1981), using the package phytools (Revell, 2012) for R. Tree visualization was performed using the Bioconductor package Ggtree (Yu et al., 2017) for R. Finally, we compared the annotation, particularly the GO Plant Slim terms, of the different topologies observed for *Ophrys* and *Gymnadenia*.

## Results

### Transcriptome Assemblies and Functional Annotation

**Figure 2:**
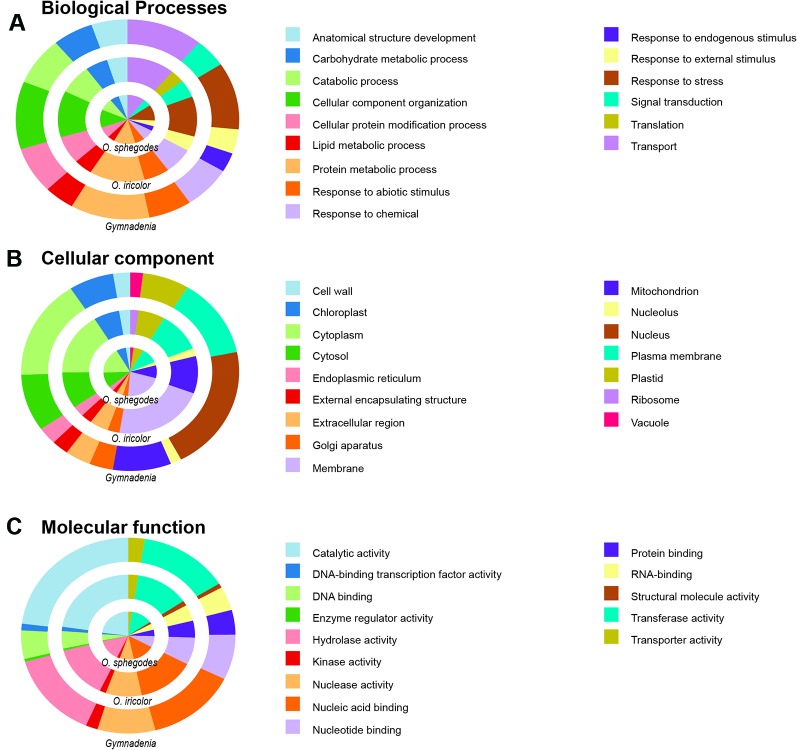
GO Plant Slim functional annotation. GO Plant Slim annotation of the three transcriptomes for the most common **(A)** Biological Process, **(B)** Cellular Component and **(C)** Molecular Function terms.

All *Ophrys* individuals were diploid (**Figure S2** for *O. iricolor s.l.*), consistent with previous studies (Xu et al., 2011; Sedeek et al., 2014). After sequencing, a total of 493.5 million paired-end (PE) reads from *O. iricolor* and 191.9 million from *Gymnadenia* were produced (**Table 1**). All the raw sequencing data (totalling 431.8 Gbp from 2111 million PE reads) are available in the Sequence Read Archive (SRA) of the National Center for Biotechnology Information (NCBI) under the accession numbers in **Table 1**. We successfully produced 131,528 and 589,218 contigs (**Table 1**) for *O. iricolor* and for the *Gymnadenia* cross-species assembly, respectively, corresponding to 88,664 and 174,633 Coding Sequences (CDSes) (**Table 2**). The remaining sequences did not match any known gene from the databases queried. The annotation tables can be downloaded from figshare (links in **Table 2**). Based on the three main Gene Ontology categories (biological process, cellular component, and molecular function), we compared the 14 most common GO Plant Slim terms (Clark et al., 2005) in the *Gymnadenia* spp. cross-species, *O. iricolor* and the updated *O. sphegodes* transcriptomes (**Table 1**, **Figure 2**). To avoid overrepresentation of general terms such as “metabolic” or “cellular” processes, we omitted the first 7, 3, and 3 terms for Biological Process, Cellular Component and Molecular Function, respectively. Overall, the three transcriptomes are very similar in GO terms. The main differences between *O. sphegodes* and *O. iricolor* are the lack of terms related to “response to endogenous stimulus” in *O. iricolor*, and the presence of terms related to “vacuole” in *O. sphegodes* (**Figures 2A, B**). On the other hand, the *Gymnadenia* transcriptome differs from the *Ophrys* transcriptome by showing a high number of genes related to “nucleus” processes and an absence of those related to "membrane" processes (**Figure 2B**). Finally, BUSCO assessments with the embryophyta lineage database indicated that the completeness of the transcriptomes was 93.4, 91.9, and 94.1% for *O. iricolor*, *O. sphegodes* v.2, and cross-species *Gymnadenia* transcriptomes, respectively (**Figure 1A**). These results therefore suggest a reasonably high assembly quality of our floral transcriptomes, especially when compared with fully sequenced orchid genomes (encoding the transcripts of all tissues), i.e. the *Apostasia* genome with a 93.62% completeness, 94.45% in *Phalaenopsis equestris* and 95.49% in *Dendrobium catenatum* (all using the embryophyta database) (Zhang et al., 2017). Also, with 87.6, 85.5, and 87% for the larger BUSCO liliopsida lineage database (**Figure 1B**), for *O. iricolor*, *O. sphegodes* v.2 and cross-species *Gymnadenia* transcriptomes, respectively, our transcriptomes appear relatively complete with respect to monocot-specific genes.

**Table 2:** Annotation statistics.

Annotation	*O. iricolor s.l.*	*O. sphegodes s.l.* v2	*Gymnadenia* cross-species
			
CDSes	88,664	167,997	174,633
BLASTX	51,706	83,722	193,416
BLASTP	75,825	126,548	275,242
Pfam	52,288	84,592	195,443
SignalP	55,429	88,779	207,377
EggNOG	57,652	92,178	211,305
KEGG	54,457	85,892	201,018
TmHMM	75,781	89,813	208,338
Gene Ontology	30,098	79,238	129,578
Figshare identifier	10.6084/m9.figshare.9944015	10.6084/m9.figshare.9944018	10.6084/m9.figshare.9944006

### Phylogenomic Analysis

**Figure 3:**
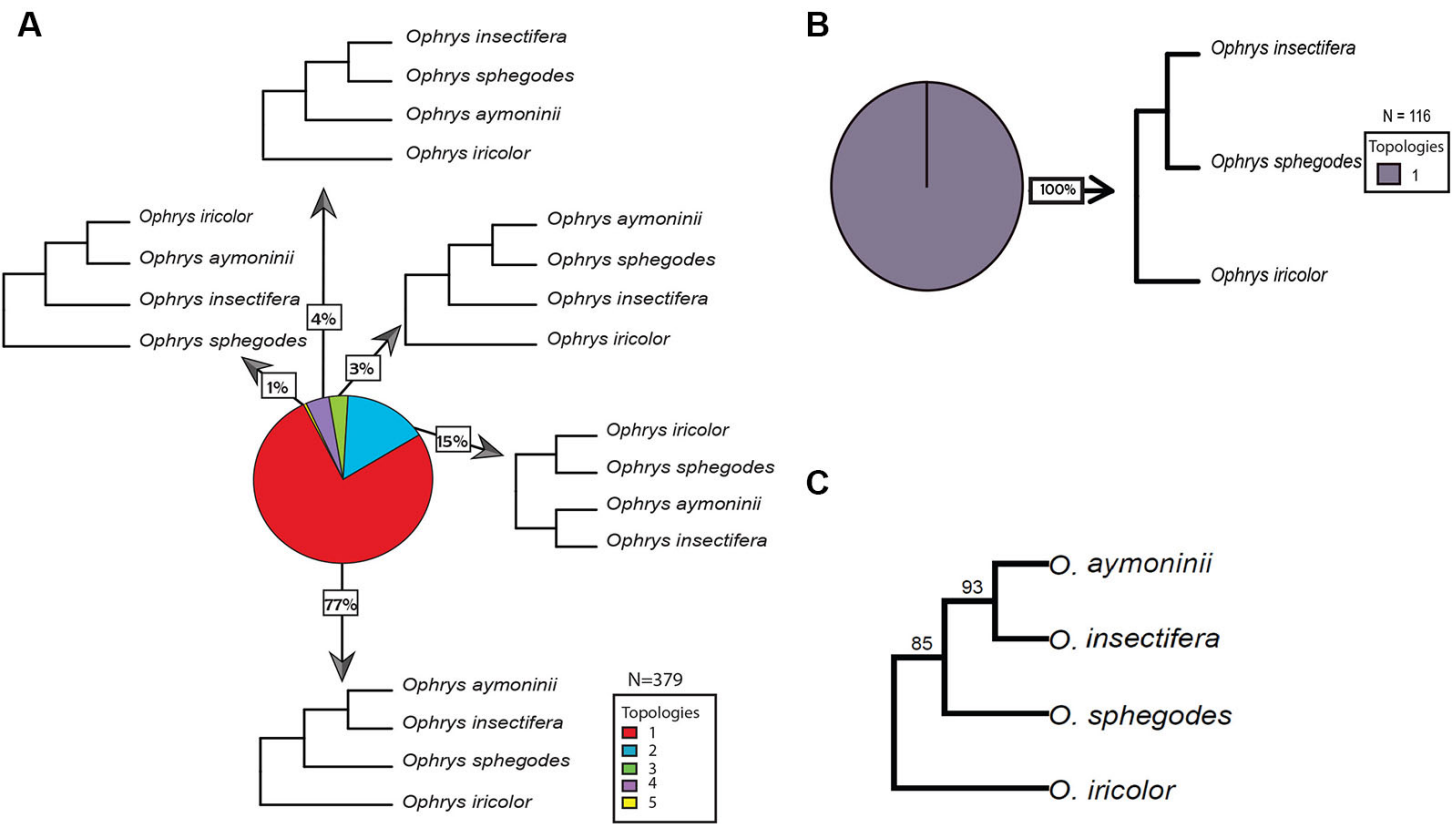
Distribution of gene tree topologies in *Ophrys*. Proportions of rooted orthologous gene tree topologies (shown without branch lengths) for *Ophrys*. **(A)** overview of four-species *Ophrys* topologies; **(B)** three-species *Ophrys* topology; **(C)** majority-rule consensus tree from all individual 4-species gene trees, where numbers above branches indicate the percentage of individual gene trees supporting a group.

**Figure 4:**
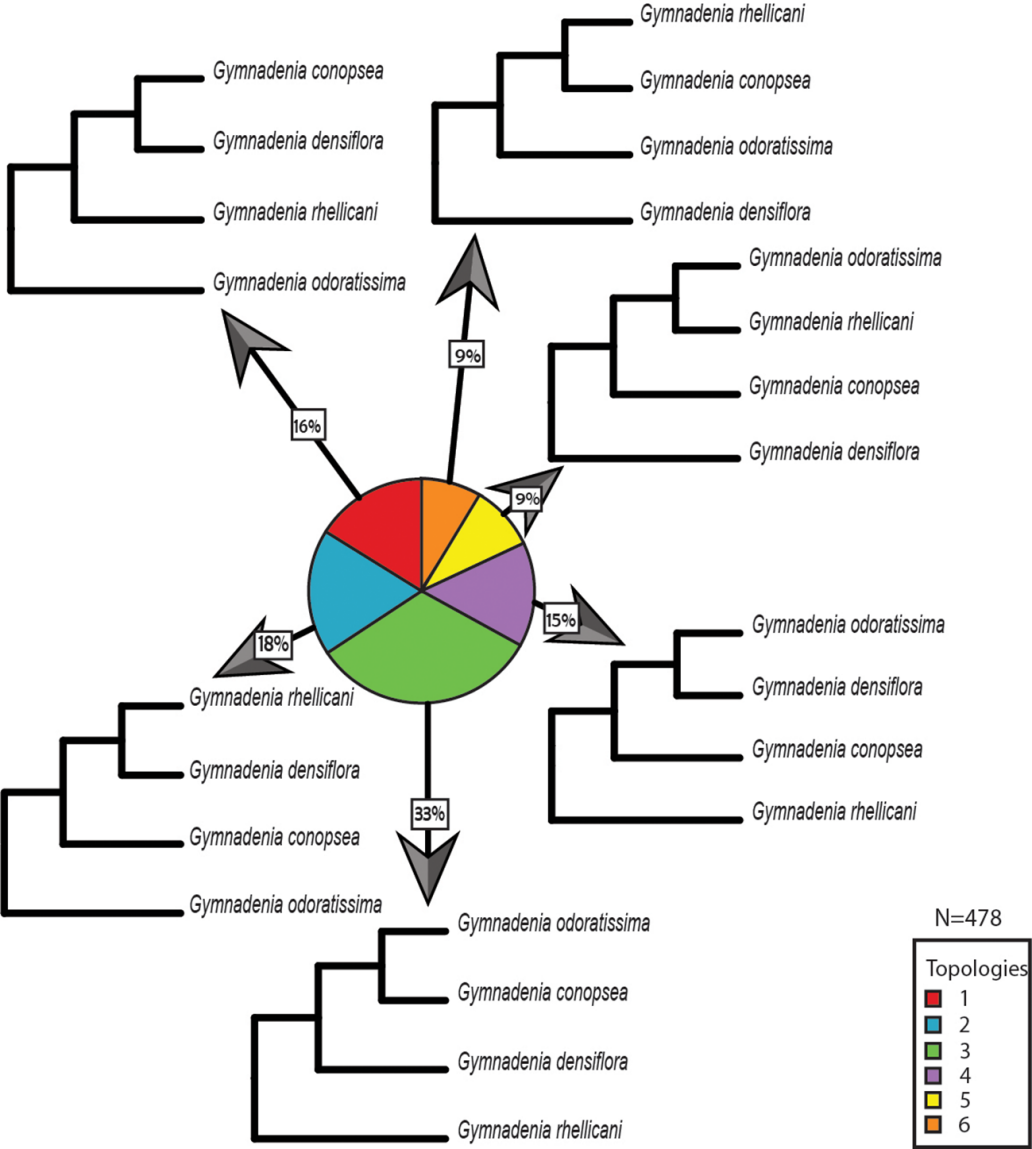
Distribution of gene tree topologies in *Gymnadenia*. Proportions of rooted orthologous gene tree topologies (shown without branch lengths) for *Gymnadenia*.

Overall, we found the 15 orchid species included in this study to share a total of 1,749 gene families. From these gene family phylogenies, 226 contain *Ophrys* monophyletic groups with no gene duplications sampled from *all Ophrys* species and 160 contain *Gymnadenia* monophyletic groups with no gene duplications sampled from *all Gymnadenia* species separately. In addition, 116 contain informative topologies with one *Ophrys* species missing; and 153 and 318 topologies contain gene duplications (or alleles) within single-species monophyletic groups for *Ophrys* and *Gymnadenia*, respectively. For *Ophrys* and *Gymnadenia*, we found 5 and 6 of the 15 possible rooted topologies for four taxa, respectively. In *Ophrys,* the most common topology (77% of the trees) suggests that *O. insectifera s.l.* (with *O. aymoninii*) is not the basalmost clade, but instead places it in a clade with *O. sphegodes,* whereas *O. iricolor* takes the basal position (**Figures 3A**, **B**). This is also evident from the consensus tree over all orthologous gene groups (**Figure 3C**) and from all (100%) of the trees missing one *Ophrys* species (**Figure 3B**). In the case of *Gymnadenia*, the distribution of topologies is more even. Yet, the most common topology, supported by 33% of the trees, places *G. rhellicani* at the basal position in the *Gymnadenia* tree (**Figure 4**). Overall, a total of 48% of evaluated *Gymnadenia* genes show a topology that places *G. rhellicani* as a sister to all other species. Also, strikingly, only 33% of gene topologies support a sister-species relationship among *G. conopsea* and *G. odoratissima*. We compared the GO annotations of each topology in *Ophrys* and *Gymnadenia*, but despite some annotation differences between topologies, there is no clear pattern with respect to putatively pollinator-relevant features (**Figures S3** and **S4**). Although not significant, the two most common *Ophrys* topologies also show the highest average branch length (**Figure S5A**), whereas two less common topologies have the longest branch lengths in *Gymnadenia* (**Figure S5B**, non-significant); these are not united by a common phylogenetic theme (e.g. with respect to *G. rhellicani*).

## Discussion

This study provides significant new transcriptome sequence resources aimed to improve our knowledge about the highly complex Orchidaceae family. Specifically, we present novel floral transcriptomes of several members of the subtribe Orchidinae of the Orchidoideae subfamily, covering both sexually deceptive and rewarding orchids. Overall, there were no striking differences between sexually deceptive and rewarding orchids when comparing the most common annotation terms based on Gene Ontology categories. This is not a surprise, because the GO Slim categories approach, although providing a large vocabulary to describe the functional categories, also suffers from a lack of clarity and too broad definitions, resulting in only a vague overview of molecular biology (Smith et al., 2003). At the same time, the phylogenetic proximity of *Ophrys* and *Gymnadenia* provides a plausible explanation for the lack of strong differentiation in terms of GO categories and suggests that differences in pollination strategy do not require fundamental changes in the genome-wide repertoire of florally expressed genes. This is in line with the phylogenetic lability of pollination strategies reported within the Orchidinae (Inda et al., 2012).

However, clear differences between *Ophrys* and *Gymnadenia* are apparent in terms of the transcriptome-wide distribution of gene tree topologies. For phylogeny reconstructions, rather than concatenating sequences, we evaluated multiple gene family trees separately. Trees derived from concatenated sequences do not reveal discrepancies between individual genes that are expected under a standard coalescent process, i.e., the more recent a species split is, the more tree topologies are expected due to incomplete lineage sorting (Takahata, 1989). Disagreement between gene trees and species trees has been observed in an increasing number of studies suggesting that the combination of a large amount of ancestral polymorphism and post-speciation gene flow between taxa can lead to large systematic differences between gene and species trees (Green et al., 2010; Novikova et al., 2016; Filiault et al., 2018; Malinsky et al., 2018).

Interestingly, the two most common gene tree topologies recovered for *Ophrys* reflect previous published phylogenetic reconstructions, our topologies 1 and 2 (**Figure 3**) corresponding to the phylogenies published most recently by Bateman et al. (2018b) and Breitkopf et al. (2015), respectively. Breitkopf et al.'s reconstruction suggested the *O. insectifera* group (clade α, including *O. aymoninii*) as the basal clade on the tree. By contrast, the phylogenetic reconstruction by Bateman et al. places *O. insectifera* closer to the *O. sphegodes* group, whereas a lineage containing the *O. fusca* complex (clade β, here represented by *O. iricolor*) is the earliest diverged. Our results, with a consensus of 85% of gene topologies, overwhelmingly support the inner placement of *O. insectifera*, rather than a basal position (**Figure 3C**). However, with the wasp-pollinated *O. insectifera* sister to the clade containing *O. sphegodes* and the wasp-pollinated *O. speculum* sister to the clade containing *O. iricolor/O. fusca*, the phylogeny's implication for the ancestral mode of pollination remains unchanged; the inference of ancestral wasp pollination in the genus *Ophrys* (Breitkopf et al., 2015) therefore seems unaffected by our findings. Nevertheless, it is striking that we found no strong evidence for discordant phylogenies throughout the genome.

Since the *O. insectifera*-group member *O. aymoninii*, a narrow endemic in southern France, is *Andrena*-pollinated (Paulus and Gack, 1990; Gervasi et al., 2017), phylogenies placing *O. aymoninii* together with the other *Andrena*-pollinated linages, *O. sphegodes* and/or *O. iricolor* could be (but need not be) an indication of hybridization and introgression via *Andrena* pollinators. Although our analysis recovers phylogenies (**Figure 3A**, topologies 3 and 5) consistent with this hypothesis, with only 4% of the gene trees overall, support for pollinator-mediated introgression is weak at best.

Unlike *Ophrys* with a clearly predominant phylogeny across the transcriptome, *Gymnadenia* presents a much less clear picture of species relationships. The sister relationship between *G. conopsea* and *G. odoratissima* has been supported in several previous studies (e.g. Bateman et al., 2003; Sun et al., 2015) including by a recent genome-wide RAD-Seq (concatenated) SNP data set (Brandrud et al., 2019). This relationship is here supported by the most common topology in the transcriptome (**Figure 4**, topology 3). Yet this is also the *only* topology that supports this relationship, accounting for only 33% of orthologous gene groups evaluated. We must therefore conclude that, from a genomic perspective, the sister relationship of *G. conopsea* and *G. odoratissima* is not beyond doubt.

The genus *Gymnadenia* now typically includes its former sister genus *Nigritella* as a subgenus. Initial hypotheses built on morphological data (Wucherpfennig, 2002), anthocyanin pigments (Strack et al., 1989), or AFLP markers (Ståhlberg, 1999) suggested the separation of the two genera. Early molecular phylogenies (usually based solely on ITS) typically sampled only *G. conopsea*, *G. odoratissima*, and a single member of *Nigritella*, which was generally the outgroup to the sister *Gymnadenia* species (Hedrén et al., 2000). When additional species were sampled and added to this basic phylogeny, *G. densiflora* (or, depending on the sampling, *G. borealis*) was shown to be the sister taxon to members of *Nigritella*, arguing for combining the genera (Pridgeon et al., 1997; Bateman et al., 1997; Bateman et al., 2003; Stark et al., 2011; Efimov, 2013). Addition of three nuclear genes did not change this topology (Rey, 2011; Sun et al., 2015). Interestingly, where authors considered multiple phylogenetic methods, conflict seems to arise in tree construction, with parsimony showing *Nigritella* as the outgroup to *G. conopsea*/*G. densiflora*/*G. odoratissima*/*G. borealis*, while Bayesian and maximum likelihood analyses demonstrate a sister relationship between *Nigritella* and either *G. borealis* or *G. densiflora* (Rey, 2011; Inda et al., 2012). In a major upgrade to the generic phylogeny, Brandrud et al. (2019) performed RAD-Seq, with contrasting results to the ITS-based phylogenies. Their phylogeny shows four *Nigritella* species as the outgroup to five *Gymnadenia* species, with no sister relation between *G. densiflora* and *Nigritella*, and the relevant nodes show high support.

Given the often contradictory results of earlier circumscription attempts, it is perhaps not too surprising that the different *Gymnadenia* gene topologies are relatively evenly distributed and that we see no single *Gymnadenia* phylogeny standing out as the best supported tree. However, the most common gene tree topology shows *G. rhellicani* as the outgroup to the other three sampled species (**Figure 4**, topology 3), in agreement with the recent RAD-Seq-based concatenated SNP analysis by Brandrud et al. (2019). Nonetheless, overall support for versus against a basal position of *G. rhellicani* is equivocal, at 48% of gene trees for (topologies 3 and 4) versus 52% against a basal position. The prevalence of other supported topologies (generally with *G. odoratissima* rather than *G. rhellicani* as the outgroup) suggests a complex population genetic history within the genus, perhaps partially due to gene exchange and incomplete lineage sorting. Neither gene annotation (**Figure S4**) nor average gene tree branch lengths (**Figure S5**) for topologies with basal *G. rhellicani* placement stand out as an indication of adaptive processes. Although *Gymnadenia* and *Nigritella* have produced one stable hybrid offspring, the apomict *G. runei* (Teppner and Klein, 1989) and other hybrids may be found, some dispute about their frequency exists (Claessens and Kleynen, 2011; Brandrud et al., 2019). Taken together, our analysis of *Gymnadenia* hints at a complex relationship among species that we are only beginning to understand. Whether this apparently more complicated pattern of genome-wide relationships in *Gymnadenia* as compared to *Ophrys* is due to the difference in pollination systems is currently unclear, although *Gymnadenia*'s less specialized pollination strategy would certainly present more opportunities for hybridization.

Using multiple gene family trees instead of one concatenated tree has proven to be a useful approach (Boussau et al., 2013; One Thousand Plant Transcriptomes Initiative, 2019). Concatenation of sequences implies that loci with a larger number of phylogenetically informative sites can bias the inference such that it may not be representative of patterns of unlinked genes throughout the genome. Also, such an approach holds no explicit information about the specific other topologies that may be useful for disentangling more complex evolutionary patterns of relationships throughout the genome, as would clearly be of interest in cases such as *Gymnadenia*. This problem is likely to be more severe in phylogenies of closely related species where excessive incomplete linage sorting may be expected and where a more sophisticated coalescent-based analysis may be valuable. Additionally, a consensus tree of individual gene trees (e.g. **Figure 3C**) is informative of the proportions of those genes in the genome that support a certain species relationship. Moreover, it is important to note that unlike a bootstrap pseudoreplicate approach, this allows for real quantification of proportions of independently segregating loci and/or functional genes and is thus more biologically meaningful. So far, our analysis only covers a small part of the genome. However, given a high-quality genome reference, future integration of this approach along chromosomes may be able to reconstruct the ancestry of individual chromosomal fragments and thereby shed light on the detailed evolutionary patterns and the role of selection (see Filiault et al., 2018) in shaping lineage divergence. The significant new sequence resources provided in this study may be a first step towards realizing this goal for European orchids in the future.

## Data Availability Statement

The datasets analysed for this study can be found in the NCBI accessions PRJNA574279 and PRJNA504609, and as indicated in **Table 1** and **2**.

## Author Contributions

Designed the project: PS. Drafted the manuscript: LP, with assistance from PS, KB, and RK. Revised the manuscript: all authors. Extracted material and prepared the libraries: JC, KS, and RK. Sequenced and processed the raw data: WQ, CA. Assembled and annotated the transcriptomes: LP, KB, JC, AR, and WQ. Conducted phylogenomic analysis: LP. Interpreted the results: LP, KB, RK, and PS. Acquired funding: KB, RK, and PS.

## Funding

This work was financially supported by the Swiss National Science Foundation (SNF) (31003A_155943 to PS). Additional support came from the University of Zurich Research Priority Programme “Evolution in Action” (PS/AR and F. Schiestl/RK), a University of Zurich Forschungskredit grant to RK, a PLANT FELLOWS Postdoctoral Fellowship grant to KB (European Union: FP7-PEOPLE-2010-COFUND Proposal 267243), and several travel grants from the Georges & Antoine Claraz Foundation Zurich (to LP, RK, KB, and PS).

## Conflict of Interest

The authors declare that the research was conducted in the absence of any commercial or financial relationships that could be construed as a potential conflict of interest.
