# Supplementary material for: A Phylogenomic Analysis of the Floral Transcriptomes of Sexually Deceptive and Rewarding European Orchids, Ophrys and Gymnadenia
Source: Front Plant Sci. 2019 Nov 29;10:1553. doi: 10.3389/fpls.2019.01553 (PMC6895147; doi:10.3389/fpls.2019.01553)
Supplement: Supplementary file 1 [file DataSheet_1.pdf]

Supplementary Figures

Figure S1. Venn diagrams of overlap in assembled transcripts (**A**) between the four *Ophrys* assemblies (**B**) between the four *Gymnadenia* single-species assemblies and (**C**) between the 15 member of the Orchidaceae family used in this study, grouped as: (i) the four *Ophrys* species as *Ophrys*, (ii) the four *Gymnadenia* species as *Gymnadenia* and (iii) the seven species *Apostasia shezhenica*, *Phalaenopsis equesteris*, *Dactylorhiza fuchsii*, *Chiloglottis trapeziformis*, *Dendrobium catenatum*, *Platanthera clavellata* and *Goodyera pubescens* as Outgroup. In this case the numbers shown are calculated as the logical `OR` of the different assemblies.

Figure S2. Ploidy analysis of *O. iricolor* and *O. mesaritica*, the ratio to internal standard of ~3 indicating diploid individuals in *O. sphegodes s.l.* (Xu *et al.* 2011; Sedeek *et al.* 2014). Samples from different locations in Crete (Kato Chorio, Phaistos, Miamou, Pirgos, Jouchtas, Pompia and Vasiliki). The final samples used for sequencing were originally from Kato Chorio, Vasiliki and Jouchtas (for *O. iricolor*) and from Pirgos (*O. mesaritica).*

Figure S3. GO Plant Slim annotation of *Ophrys* gene topologies for the most common (**A**) Biological Process, (**B**) Cellular Component and (**C**) Molecular Function terms.

Figure S4. GO Plant Slim annotation of *Gymnadenia* gene topologies for the most common (**A**) Biological Process, (**B**) Cellular Component and (**C**) Molecular Function terms.

Figure S5. Branch lengths of evaluated gene trees. Distributions of gene tree branch lengths per topology (see Figs. 3 and 4) ± standard deviation (SD) for (**A**) *Ophrys* and (**B**) *Gymnadenia*. Means are not significantly different in either genus.

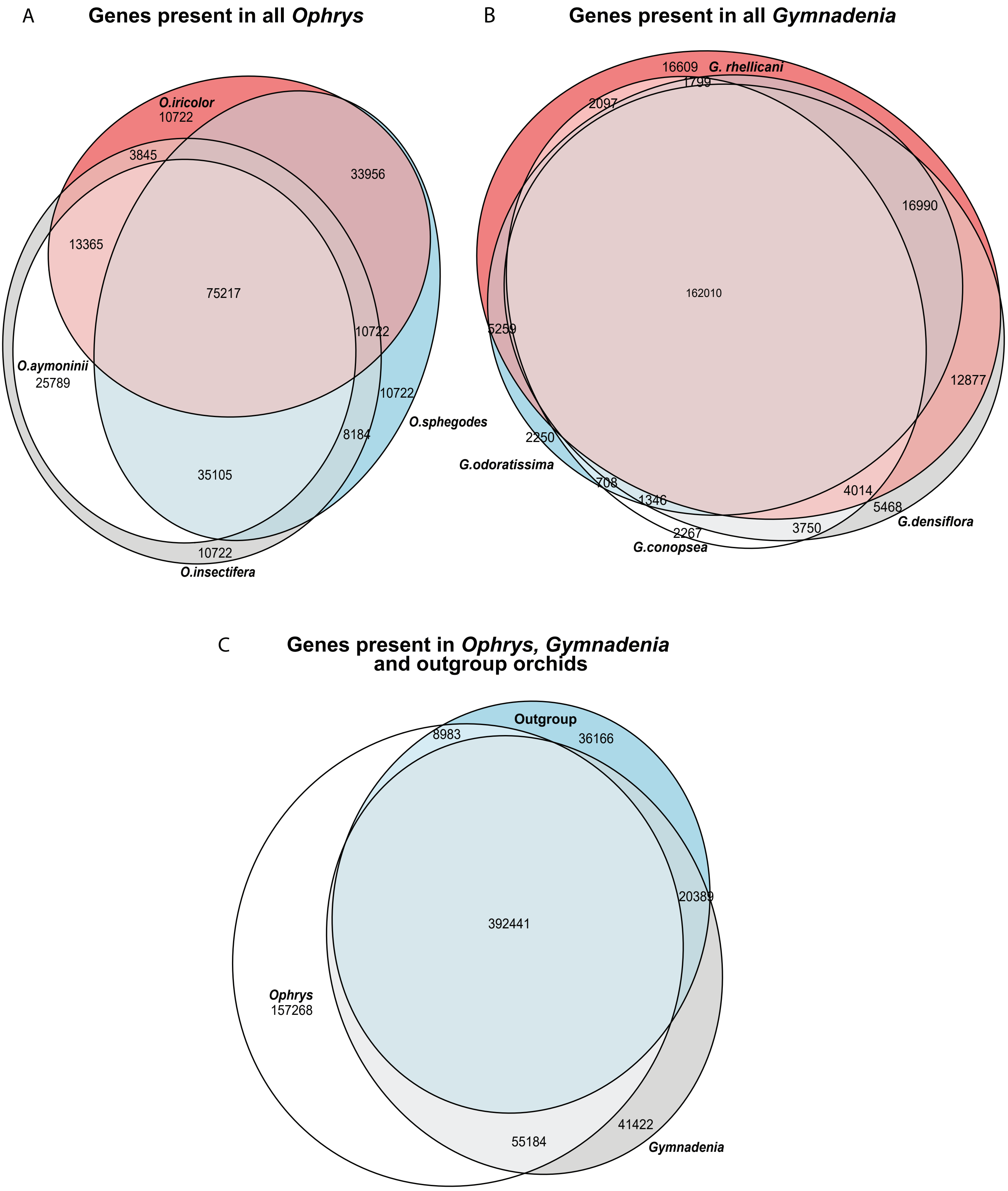
A
Genes present in all *Ophrys*
O.iricolor
10722
3845
33956
13365
75217
10722
O.aymoninii
25789
10722
O.sphegodes
8184
35105
10722
O.insectifera
B
Genes present in all *Gymnadenia*
16609
G. rhellicani
1799
2097
16990
162010
5259
12877
2250
G.odoratissima
708
1346
4014
5468
G.densiflora
2267
3750
G.conopsea
C
Genes present in *Ophrys, Gymnadenia* and outgroup orchids
Outgroup
8983
36166
20389
392441
Ophrys
157268
41422
55184
Gymnadenia

Figure S1.

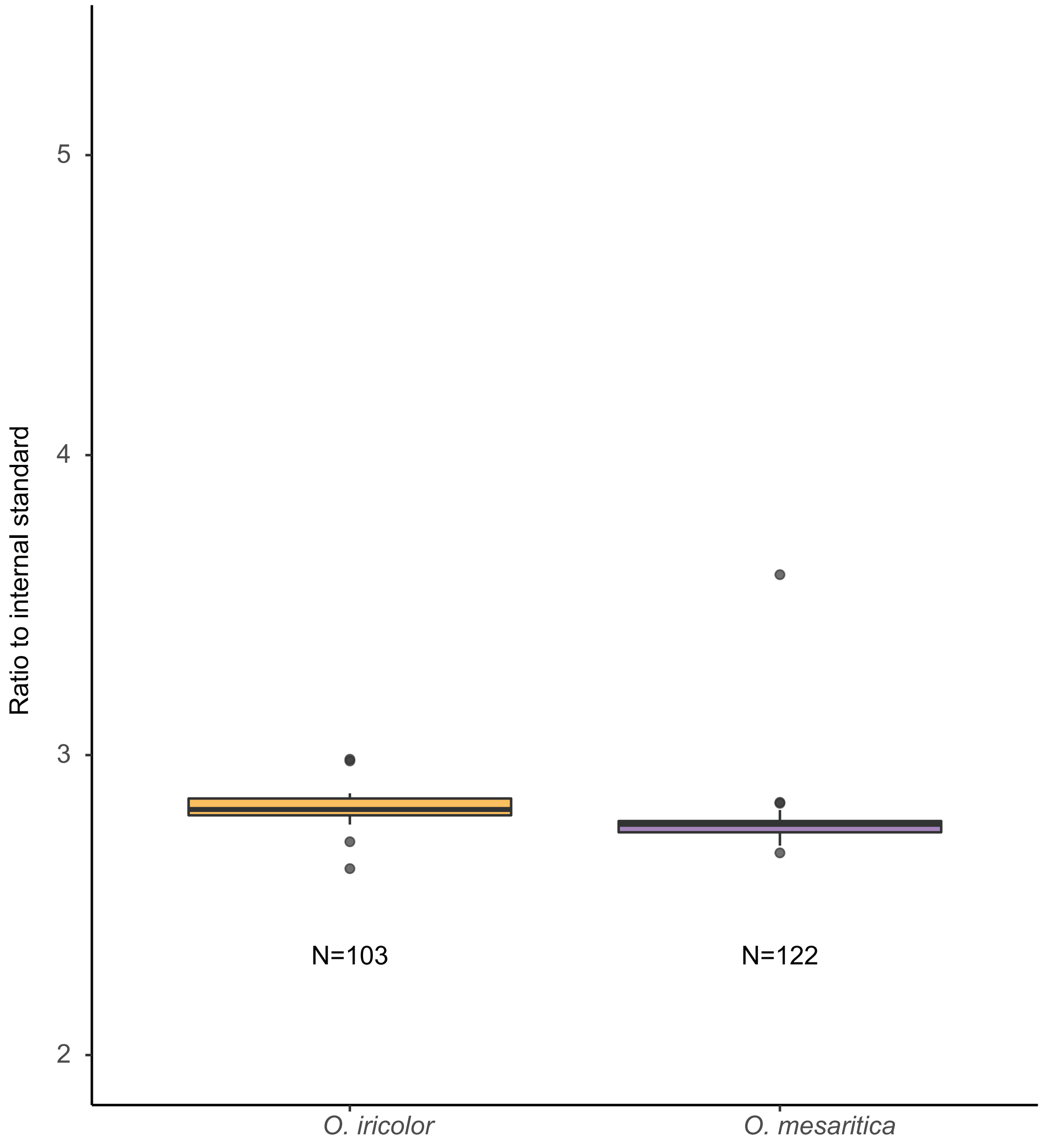
Ratio to internal standard
5
4
3
2
N=103
N=122
O. iricolor
O. mesaritica

Figure S2.

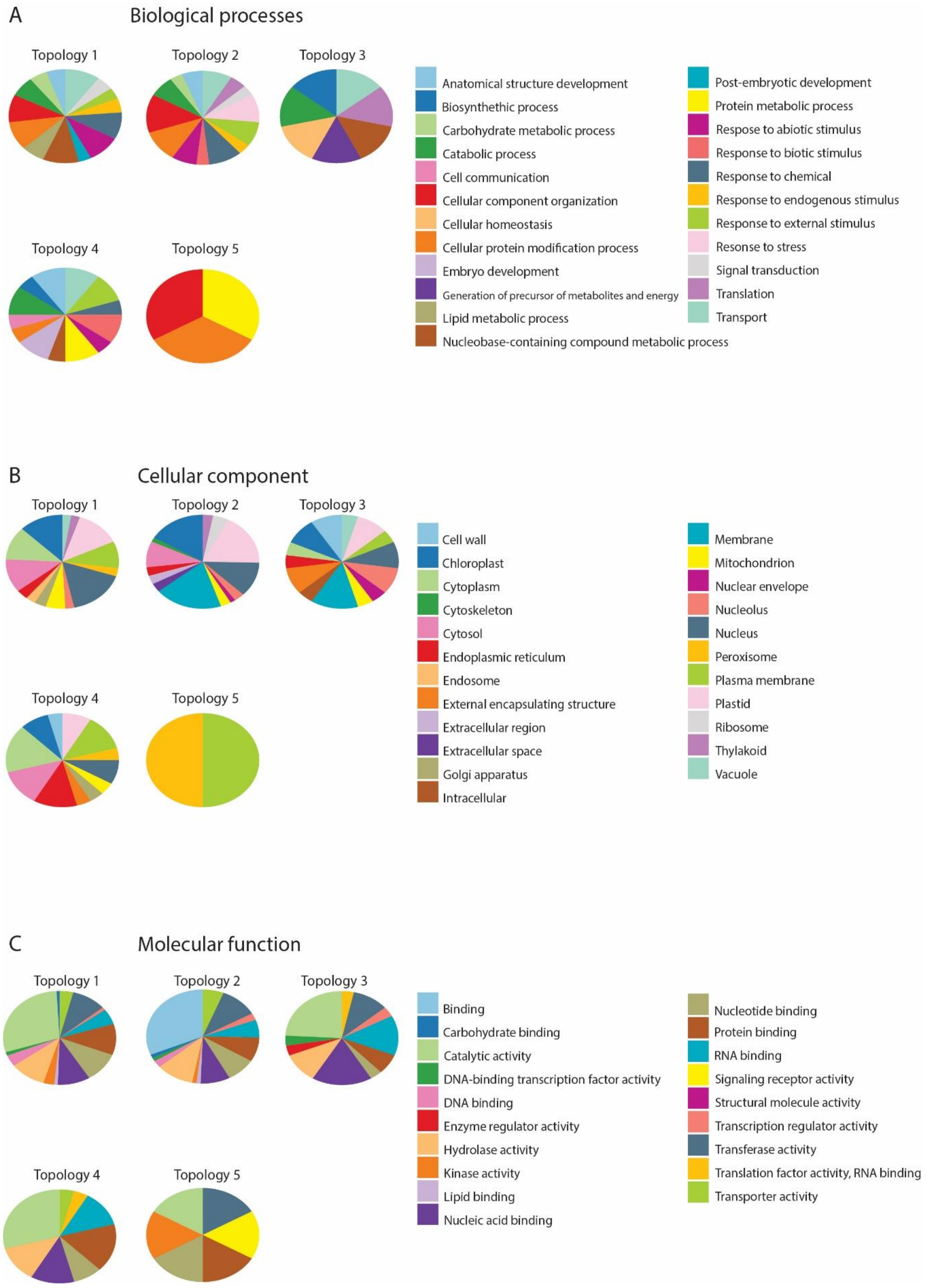
A
Biological processes
Topology 1
Topology 2
Topology 3
Topology 4
Topology 5
Anatomical structure development
Biosynthethic process
Carbohydrate metabolic process
Catabolic process
Cell communication
Cellular component organization
Cellular homeostasis
Cellular protein modification process
Embryo development
Generation of precursor of metabolites and energy
Lipid metabolic process
Nucleobase-containing compound metabolic process
Post-embryotic development
Protein metabolic process
Respose to abiotic stimulus
Response to biotic stimulus
Response to chemical
Response to endogenous stimulus
Response to external stimulus
Resonse to stress
Signal transduction
Translation
Transport
B
Cellular component
Topology 1
Topology 2
Topology 3
Topology 4
Topology 5
Cell wall
Chloroplast
Cytoplasm
Cytoskeleton
Cytosol
Endoplasmic reticulum
Endosome
External encapsulating structure
Extracellular region
Extracellular space
Golgi apparatus
Intracellular
Membrane
Mitochondrion
Nuclear envelope
Nucleolus
Nucleus
Peroxisome
Plasma membrane
Plastid
Ribosome
Thylakoid
Vacuole
C
Molecular function
Topology 1
Topology 2
Topology 3
Topology 4
Topology 5
Binding
Carbohydrate binding
Catalytic activity
DNA-binding transcription factor activity
DNA binding
Enzyme regulator activity
Hydrolase activity
Kinase activity
Lipid binding
Nucleic acid binding
Nucleotide binding
Protein binding
RNA binding
Signaling receptor activity
Structural molecule activity
Transcription regulator activity
Transferase activity
Translation factor activity, RNA binding
Transporter activity

Figure S3.

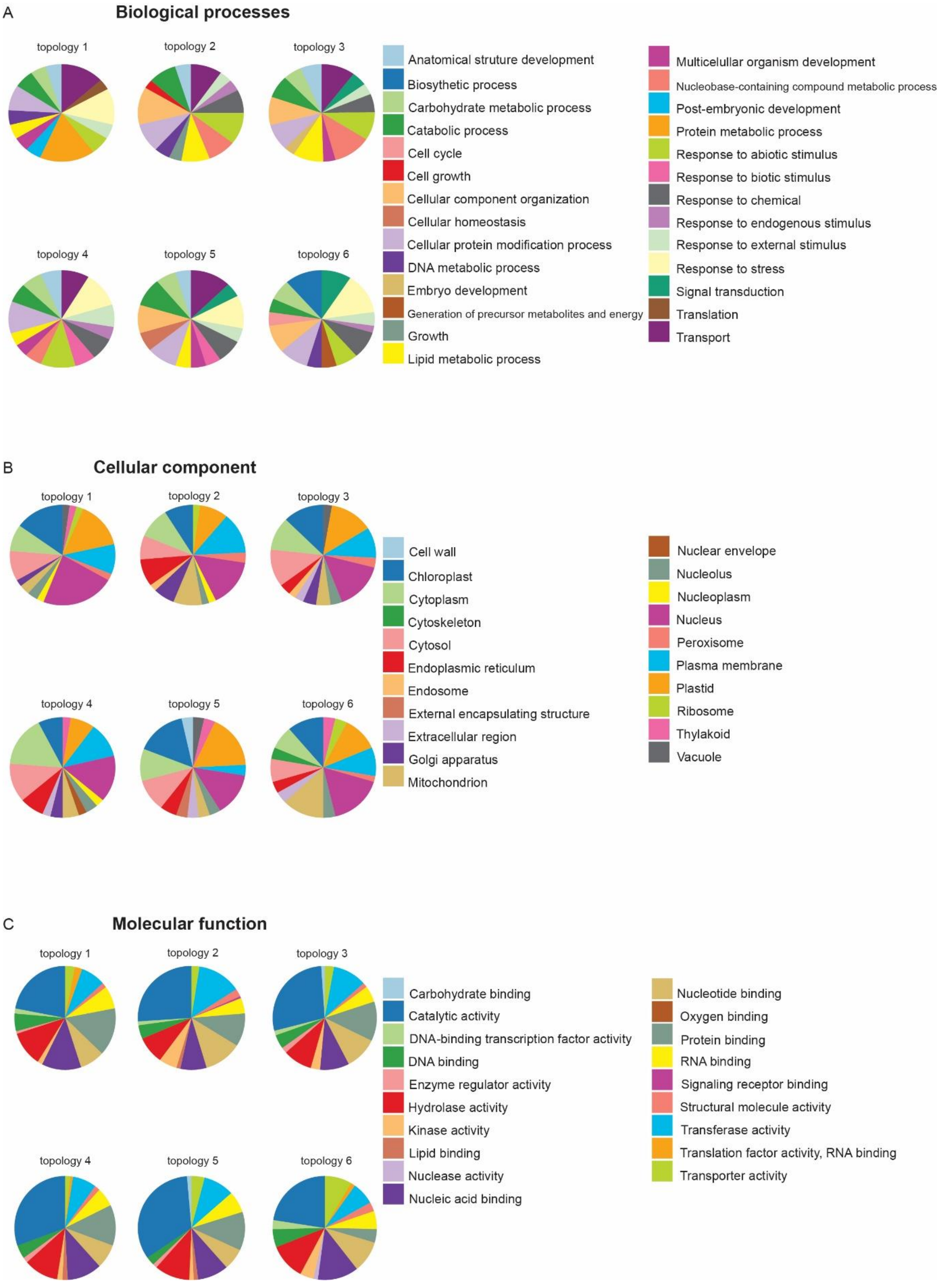
A
Biological processes
topology 1
topology 2
topology 3
topology 4
topology 5
topology 6
Anatomical struture development
Biosynthetic process
Carbohydrate metabolic process
Catabolic process
Cell cycle
Cell growth
Cellular component organization
Cellular homeostasis
Cellular protein modification process
DNA metabolic process
Embryo development
Generation of precursor metabolites and energy
Growth
Lipid metabolic process
Multicelullar organism development
Nucleobase-containing compound metabolic process
Post-embryonic development
Protein metabolic process
Response to abiotic stimulus
Response to biotic stimulus
Response to chemical
Response to endogenous stimulus
Response to external stimulus
Response to stress
Signal transduction
Translation
Transport
B
Cellular component
topology 1
topology 2
topology 3
topology 4
topology 5
topology 6
Cell wall
Chloroplast
Cytoplasm
Cytoskeleton
Cytosol
Endoplasmic reticulum
Endosome
External encapsulating structure
Extracellular region
Golgi apparatus
Mitochondrion
Nuclear envelope
Nucleolus
Nucleoplasm
Nucleus
Peroxisome
Plasma membrane
Plastid
Ribosome
Thylakoid
Vacuole
C
Molecular function
topology 1
topology 2
topology 3
topology 4
topology 5
topology 6
Carbohydrate binding
Catalytic activity
DNA-binding transcription factor activity
DNA binding
Enzyme regulator activity
Hydrolase activity
Kinase activity
Lipid binding
Nuclease activity
Nucleic acid binding
Nucleotide binding
Oxygen binding
Protein binding
RNA binding
Signaling receptor binding
Structural molecule activity
Transferase activity
Translation factor activity, RNA binding
Transporter activity

Figure S4.

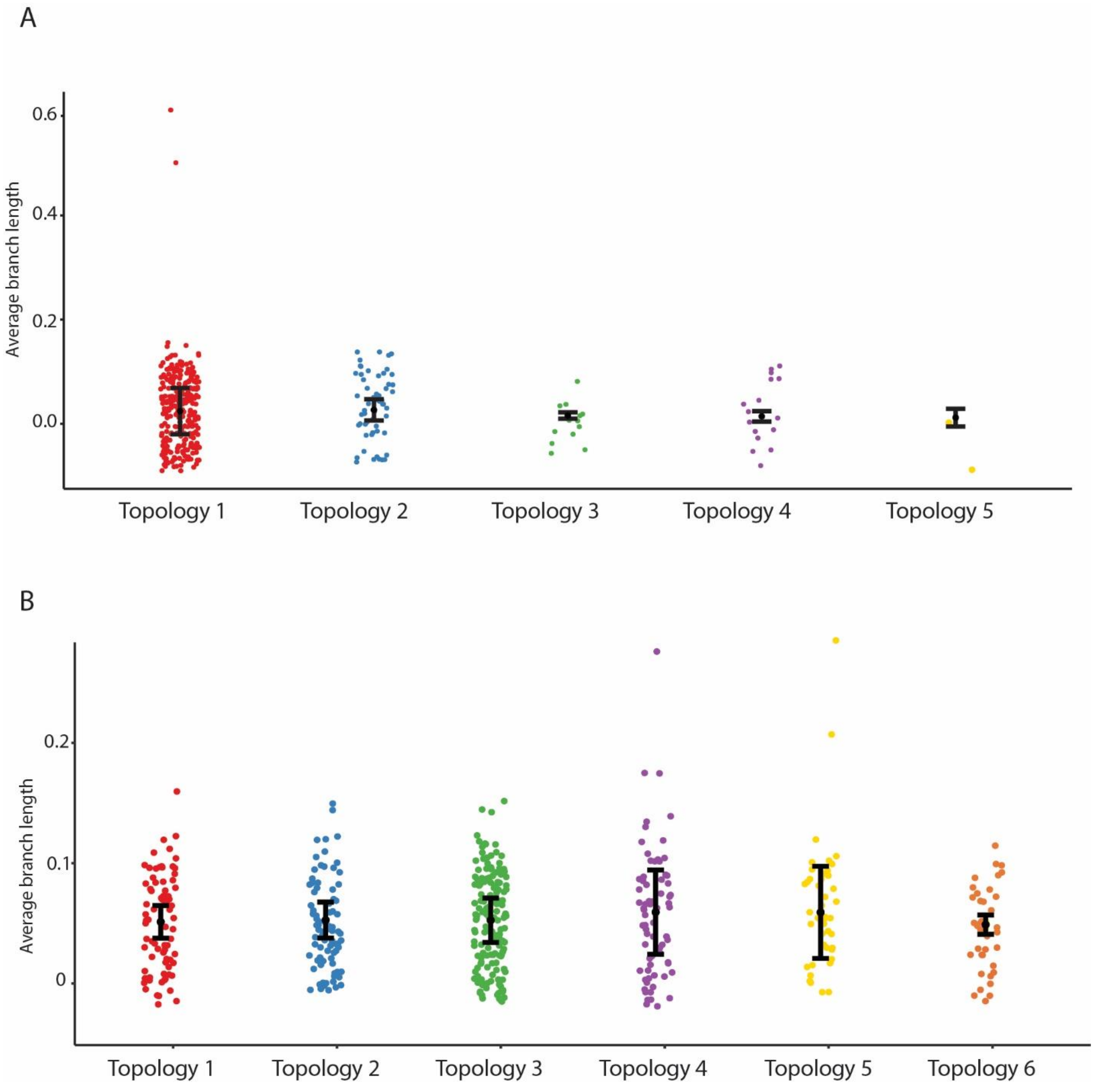
A
Average branch length
0.6
0.4
0.2
0.0
Topology 1
Topology 2
Topology 3
Topology 4
Topology 5
B
Average branch length
0.2
0.1
0
Topology 1
Topology 2
Topology 3
Topology 4
Topology 5
Topology 6

Figure S5.
